# The Potentialities of Machine Learning for Cow-Specific Milking: Automatically Setting Variables in Milking Machines

**DOI:** 10.3390/ani12131614

**Published:** 2022-06-23

**Authors:** Jintao Wang, Daniela Lovarelli, Nicola Rota, Mingxia Shen, Mingzhou Lu, Marcella Guarino

**Affiliations:** 1 Laboratory of Modern Facility Agriculture Technology and Equipment Engineering, College of Engineering, Nanjing Agricultural University, 40, Dianjiangtai Road, Nanjing 210031, China; 2018212012@njau.edu.cn (J.W.); mingxia@njau.edu.cn (M.S.); lmz@njau.edu.cn (M.L.); 2 Department of Environmental Science and Policy, University of Milan, Via G. Celoria 2, 20133 Milan, Italy; marcella.guarino@unimi.it; 3 Agribovis s.r.l., Via B. Luini 73, 20821 Meda, Italy; nicola.rota@agribovis.it

**Keywords:** algorithms, dairy cows, detachment flow rate, milking time, pulsation ratio

## Abstract

**Simple Summary:**

In dairy farms, milking-related operations and procedures are often demanding, time-consuming, and directly affect farm economics. Therefore, milking operations need to be performed efficiently and effectively, with the proper pre-dipping and post-dipping operations, and with the avoidance of overmilking. Several studies have been carried out on milking operations and the parameters for shortening milking time without harming cows. The most important prerequisites for ensuring high-level milking conditions are the appropriate pulsation ratio and detachment flow rate. Both parameters were investigated in this study, where milking operations and parameters were recorded for three months on a dairy cattle farm in Northern Italy. A comparison was made between cows milked with a pulsation ratio of 60:40 vs. 65:35 and between cows milked with a detachment flow rate of 600 g/min vs. 800 g/min. Machine learning was used to achieve automatic adjustment of pulsation ratios and detachment flows for individual cows. The least-squares support vector machine (LSSVM) classification model based on the sparrow search algorithm (SSA) applied in this study outperformed other common machine learning models. Therefore, if implemented on milking machines, this could help to automatically vary the machine’s settings based on cows’ specific characteristics, for the benefit of cows’ welfare.

**Abstract:**

In dairy farming, milking-related operations are time-consuming and expensive, but are also directly linked to the farm’s economic profit. Therefore, reducing the duration of milking operations without harming the cows is paramount. This study aimed to test the variation in different parameters of milking operations on non-automatic milking machines to evaluate their effect on a herd and finally reduce the milking time. Two trials were set up on a dairy farm in Northern Italy to explore the influence of the pulsation ratio (60:40 vs. 65:35 pulsation ratio) and that of the detachment flow rate (600 g/min vs. 800 g/min) on milking performance, somatic cell counts, clinical mastitis, and teats score. Moreover, the innovative aspect of this study relates to the development of an optimized least-squares support vector machine (LSSVM) classification model based on the sparrow search algorithm (SSA) to predict the proper pulsation ratio and detachment flow rate for individual cows within the first two minutes of milking. The accuracy and precision of this model were 92% and 97% for shortening milking time at different pulsation ratios, and 78% and 79% for different detachment rates. The implementation of this algorithm in non-automatic milking machines could make milking operations cow-specific.

## 1. Introduction

In dairy cattle farming, the milking-related operations are important and demanding since the economic benefit gained by farmers is strongly correlated to milk production [1]. Milk yield and composition are fundamental parameters for evaluating herd efficiency and herd-related management operations [2]. In Italy, the most productive dairy cow breed is the Italian Holstein, which has genetic traits aimed towards productivity and can therefore achieve very high milk yields with satisfactory fat and protein content [3]. However, the side effect is that these animals are quite delicate and require great attention to their management and health. In addition, their average lifetime is 2.2 lactations (about 4–5 years of age) [4]. A wide and comprehensive body of literature can be found on all the aspects related to milking, prediction of milk production [2], and techniques to monitor milk yield (with lactometers and software directly linked with automatic milking systems and traditional milking systems with animal identification), e.g., in [5,6,7], udder health, e.g., [8,9], welfare [10,11], feeding [12], milking operations, routine [6,13,14,15], pre-dipping and post-dipping procedures, and manual pre-stimulation by the milker, e.g., in [16,17].

Even though it is important to estimate the individual milk yield, the task is hindered by the animal being a complex time-invariant system (individuality) [18]. The availability of technology to monitor animals individually has brought significant improvements to dairy cattle farming systems, especially the introduction of automatic milking systems (AMSs) [5,12,18,19] and the subsequent possibility of making evaluations for single animals/quarters [20,21]. When traditional non-automatic milking systems are used, the milking routine substantially impacts the daily time budget of workers, and they should work carefully and efficiently for a proper milk ejection [6,22]. Pre-dipping and pre-stimulation operations are key elements for a proper milking routine and adequate oxytocin release, milk ejection, and teat health [1]. After the cluster detachment, post-dipping operations are important to avoid intramammary infections. Actions can still be carried out to shorten milking duration without damaging teat tissues. Some studies have reported that raising the b-phase of the milking vacuum by acting on the pulsation rate (60 cycles/min) or the pulsation ratio (60:40) directly and positively influences the peak milk flow rate and the milking speed [17,23]. Avoiding teat congestion and hyperkeratosis when acting on this ratio is a key element. Similarly, Bluemel et al. [24] reported on improving milk ejection and udder health quality by acting on the c-phase and d-phase. Gleeson et al. [25] and Kaskous [26] reported that small changes in the pulsation ratio (i.e., 65:35 and 67:33) do not negatively affect the teat health, while the increase of this ratio can shorten milking time and increase milk yield. Additionally, an anticipated cluster detachment can favor the preservation of teat health by avoiding high vacuum levels when the milk flow is low [1]. Although these rules are known, every farmed animal is characterized by specific biological and genetic traits that can influence their response to exogenous conditions, both during the lactation period and in the long term. Therefore, these differences need to be considered, thus reinforcing the role of precision livestock farming (PLF) techniques [27]. PLF, together with machine learning and advanced data analysis techniques, can bring continuously new advancements in the improvement of animal farming, production efficiency, and animal health and welfare status [28,29]. Suseendran and Duraisamy [30] proposed an automated model based on machine learning technology to predict the milk production of cows based on their health status, feed intake, and expected relative milk yield. Ji et al. [31] demonstrated a framework that automatically trains models using the updated farm data and predicts daily milk production, composition (fat and protein content), and frequency of milking for individual cows over the following 28 days. The application of such frameworks can be used to improve the management efficiency and animal welfare of dairy farms, also adding new knowledge that can lead to the introduction of automatic variations in milking machines. This applies mainly to contexts where farmers are not interested in AMSs but can still benefit from technology and automatization.

The goal of this study was to understand how the milking time for a herd can be reduced without harming cows’ udder health. The data on the milking operations were collected to evaluate the effect of varied parameters of the milking machine on a sample of cows. The second aim was to develop a prediction model that depends on the first 2 min of milking to automatically adjust the settings of the milking machine to a proper pulsation ratio and detachment flow rate at the individual cow level. This model can bring valuable improvements to milking operations if implemented in milking machines, because reducing the milking time of cows with high milking times can lead to an increase in the homogeneity of the herd during milking, thus improving the efficiency of milking operations.

## 2. Materials and Methods

### 2.1. Farm Description

In this study, a dairy cattle farm located in Northern Italy was monitored for three months, from September to December 2021. The herd consisted of about 1000 cows, 59% of which were primiparous and 41% multiparous. The farm was equipped with pedometers mounted on the cows’ hind legs and a milking monitoring system from the commercial firm Afimilk^®^ (Kibbutz Afikim, 1514800, Israel), from which milking data were collected for each milking session throughout the experiment period. The data collected for every cow included the milk production per session, average milking time, flow rate in the first 2 min (divided into 4 temporal sections, i.e., 0–15, 15–30, 30–60, and 60–120 s), and peak and removal flows and times. Furthermore, the gynecological state, age, parity, and days in milking (DIMs) were also accessible.

The cows were housed in a loose housing system and fed on a mixed ration. Two milking sessions per day (8 a.m. and 8 p.m.) were performed by two groups of milkers (each group was composed of 2 milkers and 1 worker that moved the animals from the farming area in the barn to the holding area). The milking took place in a traditional herringbone milking system with 15 + 15 stalls. The herd was divided into 9 groups based on herd management criteria, of which 1 group included only cows that just calved. In contrast, the others included primiparous or multiparous dairy cows. The whole milking operation lasted about 9 h daily. Both milkers carried out pre-dipping and post-dipping operations in both milking sessions, which was a good procedure for the milk ejection and udder health but increased the duration of milking [6]. During pre-dipping, the milkers stimulated and cleaned teats; they eliminated the first ejected milk, applied a detergent, and then cleaned the teats with dedicated paper; finally, they attached the cluster. About 1.5–2.0 min was the time required between stimulation and cluster attachment. The first milker worked on the first 8 cows, while the second milker on the other 7 cows in each line of stalls. After milking, post-dipping operations were completed. The milking machine was set to a pulsation ratio equal to 60:40, a vacuum pressure of 42 kPa, and the detachment of liners at 600 g/min, which was compliant with the suggested values reported in the literature [13]. Triangular liners were used and replaced when needed. Given the significant amount of time required for milking operations, the farm manager asked that the duration of milking operations be reduced, without the willingness to adopt an AMS. The attention was therefore paid to those cows that were slow to eject milk (average milking time > 8 min) and thus prolonged the whole duration of milking operations; moreover, attention was also paid to the possibility of modifying the detachment flow rate of the milking machine (from 600 to 800 g/min) to avoid the risk of overmilking and possibly further reduce milking time. The low-flow period could last longer if the set detachment threshold was not achieved.

### 2.2. Experimental Setup

The whole herd was submitted to a trial where some parameters in the milking operations varied, and the udder health was monitored. In particular, the full set of data that could be collected with the informatization of milking operations was available, including cow ID, milk yield (kg/session), average milking time (minutes), and the flow rate in the first 2 min (g/min), and split into 4 groups (0–15, 15–30, 30–60, and 60–120 s), peak flow (g/min), peak time (min), and removal flow (g/min) per each session of milking as well as the average milk yield of the previous 10 days. The removal flow did not identify the instantaneous flow at the detachment of the cluster, but it was the average flow measured for some seconds before the detachment.

With respect to the udder health, in the same period in which the milking was monitored, an expert observed the udder health through the teat-end score (TS) test every 3 weeks to evaluate possible damages to teats. In total, 5 observations were performed for the herd, renamed with the TS code from 1 to 5 (TS-1 to TS-5) in chronological order. A score from 1 to 4 was given to each teat of the udder based on its hyperkeratosis level: 1 for normal teats, 2 for smooth, 3 for rough, and 4 for very rough [32]. This operation was carried out concurrently with the morning session of milking. Additionally, the somatic cell count (SCC) was determined every month for each cow after the analysis of milk samples. Finally, the possible presence of clinical mastitis in the experimental period was investigated to determine if some cows had more clinical mastitis than others.

For this experiment, 2 trials were performed in which the pulsation ratio or the detachment flow rate was changed. The trials were carried out simultaneously, with one group of cows dedicated to the first trial and another group to the second trial. No temporal or managerial difference needed to be considered because the animals’ management and routine were the same for both trials. The selection of cows was carried out according to average days in milk (DIMs); since the experiment lasted 3 months, cows with DIM > 200 were excluded from the random selection to prevent them from being dried off before the end of the experiment. A second restriction was included in the trial on the pulsation ratio because only those cows with an average milking time (AMT) longer than 8 min were included in this trial. This selection was applied because reducing the AMT of cows that had AMT > 8 mins made the whole milking operation more homogeneous and finally shorter. Based on these prerequisites, the following 2 trials were carried out:

- Trial 1—Pulsation ratio: from the list of cows with an average milking time (AMT) > 8 min, 54 cows were randomly selected for the trial group, and the pulsation ratio was set to 65:35 (noted as Trial 1—puls 65:35); the control group including 54 cows was kept at 60:40 (noted as Trial 1—puls 60:40).
- Trial 2—Detachment: from the remaining dairy cows of the herd, a group of 60 cows was randomly selected and set to a detachment (or removal) flow of 800 g/min (Trial 2—detach800), while the control group including 60 cows was maintained with a removal flow set to 600 g/min (Trial 2—detach600).

The cows selected for both trials had, on average, a DIM of 93 and parity of 1.6. Therefore, changes during the lactation period were not affected by the differences in DIM or parity. To determine the conditions of the herd before the start of the trial, the measured SCC was calculated as a linear score, and the average of all the tested animals was equal to 2.67 ± 2.09 linear score (logarithmic linear scale based on the number of cells/mL).

Descriptive statistics on the data collected were performed with the software SAS 9.4. Moreover, multivariate statistics were also carried out to test the trials and the statistical significance. A mixed procedure (proc mixed) was carried out to predict the variables of AMT, milk yield, total low flow, peak time, peak flow, and removal flow. A random statement was introduced for the cows and a repeated statement for the repetitions of measurements, i.e., 2 milkings per day. Corrected least-square means (LSMs) and statistical differences with Tukey test were calculated between the tested trials. In particular, LSMs were calculated separately for Trial 1—puls60:40 vs. puls65:35 and Trial 2—detach600 vs. detach800. As mentioned above, the reason for the analysis being carried out separately was that the two trials were not interconnected. Then, a GLIMMIX procedure (proc GLIMMIX) was used to evaluate if statistical differences could be found within the trials with respect to the health status determined by the measurements of TS and SCC. Additionally, in this case, the Tukey test was used.

### 2.3. Prediction of Individual Cow Milking Times and Removal Flow Based on SSA-LSSVM

This study aimed at automizing the changes in the settings of the milking machine for individual cows, based on data of the average milk yield for the previous 10 days and flow rates of 0–15, 15–30, 30–60, and 60–120 s. Through this, automatic adjustment of milking parameters for individual cows and improved milking efficiency and farm welfare can be achieved. Hence, if the individual cow’s milking time can be predicted, it is possible to reduce milking time by adjusting the pulsation ratio for cows with high milking time. Moreover, if individual cow removal flow rates can be predicted, milking time can be reduced by adjusting the removal flow threshold for individual cows with too low a removal flow rate. By processing the collected data, the prediction of the individual cow milking times and removal flows will allow a precise milking parameter customization for individual cows.

#### 2.3.1. Data Normalization

Data normalization is an essential preprocessing technique used to prevent wide variation in the data, which can influence the predictive abilities of the developed system. Therefore, a mean normalization approach was adopted in this study. A reclassification was performed for the different parameters using the following formula (Equation (1)).

(1) $$X_{N,i}=\frac{X_{i}}{\bar{X}}$$

where $X_{N,i}$ is the normalized parameter of sample i, $X_{i}$ is the original parameter of sample i, and $\bar{X}$ is the average of all parameters.

#### 2.3.2. Least-Squares Support Vector Machines (LSSVMs)

A support vector machine (SVM) is a type of generalized linear classifier that is used to classify data in a supervised learning manner, where the decision boundary is the maximum-margin hyperplane over the learned samples and is part of statistical learning theory. Although SVM has a strong generalization capability, its inequality function is complicated. Suykens and Vandewalle [33] proposed a least-squares support vector machine (LSSVM) algorithm that uses a least-squares linear system as a loss function to change the inequality constraint into an equation constraint by letting the training sample be N {(x1, y1),( x2,y2) …(xi, yi)} where x, y are the n-dimensional input and output of the training sample, respectively, and given the LSSVM objective optimization function J, as shown in Equation (2)

(2) $$J\left(w,e\right)=\frac{{\zeta w}^{T}w}{2}+\frac{C}{2}\overset{N}{\underset{i=1}{\sum }}e_{i}^{2}$$

where ζ is the controllable parameter, w is the weight vector, and C is the regularization parameter used to fit the error; $e_{i}$ is the error, which needs to satisfy Equation (3).

(3) $$y_{i}=w^{T}\phi \left(x_{i}\right)+b+e_{i},\;i=1,2,\ldots ,N$$

where $\phi \left(x_{i}\right)$ is the mapping function and b is the bias value; then, the Lagrangian function is given by Equation (4).

(4) $$L=J\left(w,e\right)-\sum_{i=1}^{N}\alpha_{i}\left[w^{T}\varphi \left(x_{i}\right)+b+e_{i}-y_{i}\right]$$

where $\alpha_{i}$ is the Lagrangian multiplier, with the derivative yields (Equation (5)):

(5) $$\{\begin{array}{c} \frac{\partial L}{\partial w}=0\to w=\overset{N}{\underset{i=1}{\sum }}\alpha_{i}\varphi \left(x_{i}\right),\\ \frac{\partial L}{\partial b}=0\to \overset{N}{\underset{i=1}{\sum }}\alpha_{i}\varphi \left(x_{i}\right)=0,\\ \frac{\partial L}{\partial e_{i}}=0\to \alpha_{i}=\zeta e_{i,}\\ \frac{\partial L}{\partial \alpha_{i}}=0\to w^{T}\varphi \left(x_{i}\right)+b+e_{i}-y_{i}=0 \end{array}$$

Solving the linear equation, eliminating ω and e, gives Equation (6).

(6) $$\left[\begin{array}{c} 0\\ 1_{m} \end{array}\;\begin{array}{c} 1_{m}^{T}\\ \Omega +I_{N}/C \end{array}\right]\left[\begin{array}{c} b\\ a \end{array}\right]=\left[\begin{array}{c} 0\\ y \end{array}\right]$$

where $I_{N}$ is the n-dimensional unit matrix, 1 m =[11, 12, 13, …, 1m] is a vector of ones, and Ω is calculated by the following formula from Mercer’s theorem as given in Equation (7).

(7) $${\;\Omega }_{\mathrm{ij}}=\varphi {\left(x_{i}\right)}^{T}\varphi \left(x_{j}\right)=K\left(x_{i,}x_{j}\right)$$

Then, the decision function y(x) of the LSSVM is obtained as in Equation (8).

(8) $$\;y\left(x\right)=\mathrm{sgn}\left[\sum_{i=1}^{N}\alpha_{i}K\left(x_{i},x_{j}\right)+b\right]$$

Additionally, the radial basis function $K\left(x_{i},x_{j}\right)$ is given by:

(9) $$\;K\left(x_{i},x_{j}\right)=\exp\left(\frac{-\frac{1}{2}∥x_{i}-x_{j}{∥}^{2}}{\sigma^{2}}\right)$$

where σ is the kernel function width factor, and $∥x_{i}-x_{j}{∥}^{2}$ is the squared Euclidean distance between two vectors.

#### 2.3.3. SSA-LSSVM

The sparrow search algorithm (SSA) is a novel swarm intelligence optimization algorithm proposed by Xue and Shen [34]. It is a heuristic algorithm that mimics the cooperative behavior of a flock of sparrows during foraging to improve the exploration and use of values of parameters in the optimal search space. Since the parameter significantly influences the prediction performance of the LSSVM algorithm, it needs to be optimized based on the SSA algorithm, which can address the problem of blind selection of the parameters and the difficulty of jumping out of local extremes.

#### 2.3.4. Quantification of Prediction Model Performance

A five-fold cross-validation strategy with 10 repetitions defined by Siegmann and Jarmer [35] was applied to evaluate the classification performance of all the tested machine learning algorithms based on accuracy (Equation (10)), precision (Equation (11)), recall (sensitivity) (Equation (12)), and the F-measure (Equation (13)).

(10) $$\;\mathrm{Accuracy}\;=\frac{\left(\mathrm{TP}\;+\;\mathrm{TN}\right)}{\;\left(\mathrm{TP}\;+\;\mathrm{TN}\;+\;\mathrm{FN}\;+\;\mathrm{TN}\right)}$$

(11) $$\mathrm{Precision}\;=\frac{\mathrm{TP}}{\left(\mathrm{TP}\;+\;\mathrm{FP}\right)}$$

(12) $$\mathrm{Recall}\;=\frac{\mathrm{TP}}{\left(\mathrm{TP}\;+\;\mathrm{FN}\right)}$$

(13) $$\;F-\mathrm{measure}\;=2\times \frac{\left(\mathrm{Precision}\;\times \;\mathrm{Recall}\right)}{\left(\mathrm{Precision}\;+\;\mathrm{Recall}\right)}$$

where TP is the number of true positives, TN is the number of true negatives, FP is the number of false positives, and FN is the number of false negatives.

## 3. Results and Discussion

### 3.1. The Effect of Pulsation Ratio and Removal Flow on Milking Characteristics

The use of the mixed model to predict the variables of AMT, milk yield, total low flow, peak time, peak flow, and removal flow allowed us to achieve interesting insights into the studied herd. The results with statistical differences for Trial 1 (puls 60:40 vs. puls 65:35) and Trial 2 (detach 600 vs. detach 800) are reported in Table 1 and Table 2, respectively.

Regarding the trial performed for the pulsation ratio (Trial 1), from the results it was found that there was no statistically significant difference in the milk yield, peak flow, or removal flow when the pulsation ratio was 60:40 compared to 65:35 (*p* > 0.05). Instead, milking time (AMT) was significantly reduced, from 8.42 min to 7.97 min (*p* < 0.0001); the peak time was significantly reduced, with a decrease of 7%, and the total low flow increased significantly, from 1.10 to 1.40 min. Milk yield increased slightly but not significantly in this sample (from 18.8 to 19.2 kg per session). Therefore, with the modification of the pulsation ratio from 60:40 to 65:35, the goal of reducing milking time was achieved. These results concur with studies on milking operations which concluded that the pulsation ratio needs to be varied so as to reduce the milking time [17,25,26]. However, as suggested by Kaskous [26], the reduction in milking time while increasing the pulsation ratio may compromise the udder health. Therefore, every change in the machine settings needs to be investigated in detail.

In Trial 2, the two different detachment flows were tested, which resulted in the differences shown in Table 2. No statistically significant difference was found in the AMT, peak time or peak flow between the experimental group with a detachment equal to 800 g/min and the control group with a detachment of 600 g/min (*p* > 0.05). Milk yield was statistically significantly lower (*p* < 0.05) (18.5 and 17.8 kg in detach600 and detach800, respectively), as well as the total low flow (0.76 and 0.64 min in detach600 and detach800, respectively). The removal flow significantly improved (*p* < 0.001) from 1.0 kg/min to 1.2 kg/min. Stauffer et al. [36] found no differences in milk production when the cluster detachment flow changed, similarly to the findings of this study. This similarity was achieved even if Stauffer et al. [36] used different detachment thresholds than in this experiment. Moreover, Besier and Bruckmaier [37] stated that the earlier cluster detachment can slightly reduce milk yield with the benefit of milking time and teat health. Their study concluded that the best removal flow would be at least 600 g/min, which is consistent with this experiment.

Furthermore, in the literature, many researchers highlight the relevance of the vacuum pressure, showing that values around 43 kPa are optimal for both milk ejection and teat health. However, in this study the vacuum pressure was not changed.

### 3.2. The Effect of Pulsation Ratio and Removal Flow on SCC, Mastitis Incidences, and TS

To evaluate if the tested parameters influenced the udders’ health, the somatic cell count (SCC), expressed as a linear score, and the teat-end score (TS) were measured, and the statistical difference for each group was established. As reported in Atakan et al. [38], it is important to monitor the TS because hyperkeratosis on the teat end is caused mainly by errors, high vacuum pressure of the milking machine, increased milk yield, prolongation of milking, dirtiness of the animals, and insufficient bedding. Therefore, reducing the milking time may help to reduce the TS of cows with a high average milking time. However, the higher vacuum pressure and other characteristics that were not monitored in this experiment (e.g., dirtiness and bedding) could also affect this result. Additionally, the TS is also important due to its relationship with a higher risk for mastitis and high SCC [39]. However, as stated by Sharma et al. [39] there are several reasons for a high concentration of SCC.

As a first consideration, the SCC was calculated as a linear score, so that values above 4 mean that the SCC was excessively high (>300,000 cells/mL). SCCs were also measured before the start of the trial, with the SCC-0 measurement referring to the measurement of SCC before the start of the trial. SCC-1, SCC-2, and SCC-3 refer to the three measurements taken during the experiment.

In the statistical analysis carried out with the GLIMMIX procedure, SCC-0 was included in the model, and SCC-1, SCC-2, and SCC-3 were predicted. From the model outcomes, the results shown in Table 3 and Table 4 were achieved. In particular, in Trial 1 (shown in Table 3), SCCs were always higher when the pulsation ratio was set to 60:40; only in the second measurement was this difference not significant (*p* > 0.05). In the three observations, the LSMs were below the threshold of 4, meaning somatic cells were <300,000/mL.

Running the same model for Trial 2, the SCC of detach600 was always significantly different from detach800. In SCC-1, the linear score was lower for detach600, while in SCC-2 and SCC-3 it was significantly higher (*p* < 0.0001). Furthermore, in this case, it must be highlighted that the SCC_3 was above 4, which corresponded to an SCC > 300,000/mL.

Considering the TS, five measurements were carried out in the experimental period, from September (TS-1) to December (TS-5). According to the classification by Mein et al. [32], a teat-end score from 1 to 4 was given to each teat of the udder based on its hyperkeratosis level. Each TS was averaged for the front teats and for the back teats of each cow.

Table 5 shows the teats’ scores, averaged for front and back teats, for the experimental and control groups in the two separate trials. The mean TS was higher in the experimental group than in the control group in both trials. Teats showed a general increase in the hyperkeratosis with the progress of the experiment. Moreover, the TS of the back teats showed less damage than the front ones. However, fluctuations were observed. According to the data and the incidences of clinical mastitis, the reason for the fluctuations was due to some cows having developed serious mastitis in the period of the previous TS test, and after the antibiotics treatment, cows had a lower score.

Considering that the cows in Trail 1 were randomly selected from a herd with an average milking time higher than 8 min, it could be inferred that the longer milking time negatively influenced teat health, as also suggested by Odorčić et al. [1].

In order to understand the statistical significance of these differences, the GLIMMIX model was carried out for the different TS. The results are reported in Table 6 for Trial 1, and in Table 7 for Trial 2.

In Trial 1, differences between the puls60:40 and puls65:35 were all significant. In all cases, the TS was higher in puls65:35 than puls60:40.

Regarding Trial 2, in all cases statistical differences were present between detach600 and detach800, with a better TS achieved when detaching the cluster at 600 g/min instead of 800 g/min.

**Figure 1:**
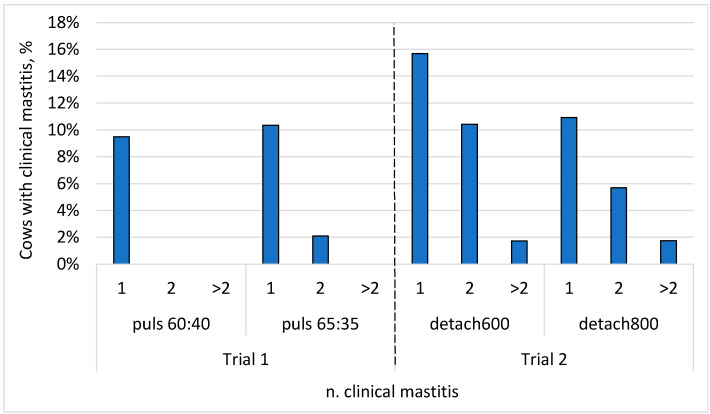
Percentage of cows with clinical mastitis infection during the experimental period, distinguished between Trial 1 (puls60:40 vs. puls 65:35) and Trial 2 (detach600 vs. detach800). The number of cases of clinical mastitis was 1, 2, or >2.

Concerning the clinical mastitis, Figure 1 shows the frequency of incidence of clinical mastitis during the tested trials. In all the cases, most of the cows were not infected during the experimental period. In Trial 1, 9–10% of cows had one case of clinical mastitis; in Trial 2, between 11 and 16% of cows encountered one case of clinical mastitis, with the control trial showing a higher frequency. Only 2% of cows had >2 cases of clinical mastitis, all in Trial 2.

### 3.3. Prediction of AMT and Removal Flow Based on SSA-LSSVM

The test environment for this study was CPU core i5-1135G7, 2.4 GHz, 16 G RAM, programmed with MATLAB R2019a (The MathWorks Inc., Natick, MA, USA). To compare the prediction performance of SSA-LSSVM with other machine learning models, i.e., the K-nearest neighbor (KNN), naive Bayes, decision tree, and linear discriminant analysis (LDA) were trained. Based on the above model, binary predictions were made for AMT (≥8 min and <8 min) and for removal flow (≥600 g/min and <600 g/min).

One thousand randomly selected samples were used to test the prediction performance of each model. Table 8 and Table 9 show the evaluation metrics of each model for the AMT prediction model and the removal flow prediction model, respectively. As shown in Table 8, it can be observed that SSA-LSSVM achieved an accuracy equal to 92% and had the highest F1 score of 0.95, which makes it the best predictor of AMT. Additionally, Table 9 presents that the removal flow prediction test results of SSA-LSSVM had the best performance, with the highest prediction accuracy (78%) and the highest F1 score (0.88). Based on the evaluation metrics of the model, it can be concluded that the prediction model developed is satisfactory for AMT and acceptable for removal flow. In addition, the same model had different predictive abilities for different objects. For example, LDA, which is only second to SSA-LSSVM in predicting AMT, was the worst among several models in predicting removal flow. From both Table 8 and Table 9, it can be observed that the LSSVM model optimized by SSA had a better accuracy, precision, recall, and F1 score than the unoptimized model (with no SSA). Therefore, the optimization of the LSSVM using the SSA algorithm was valid and effective.

#### Model Performance with Dimensionality Reduction

**Figure 2:**
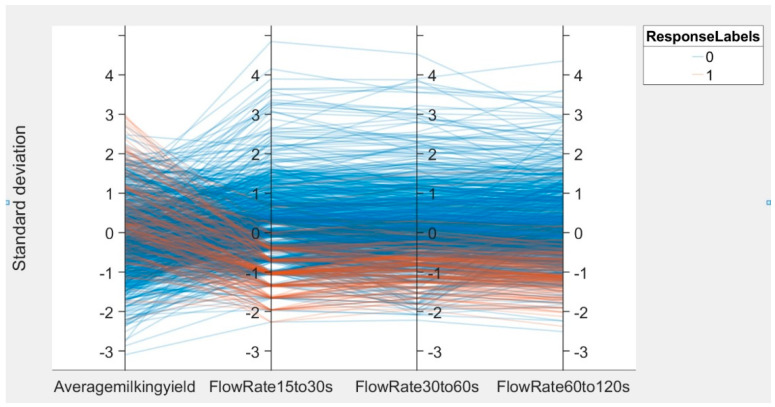
Feature parallel coordinate plot.

To determine the predictive power of the data variable, a dimension reduction technique was performed, and the performance of the SSA-LSSVM was evaluated (based only on the high predictive power variables). Figure 2 presents a parallel coordinate plot of the 1000 test samples after data normalization. It can be observed that the three features, ‘flow rate 15–30′, ‘flow rate 30–60′, and ‘flow rate 60–120′, were more centrally and distinctly classified and thus had a high predictive power. Therefore, only these three features were used to retrain the two prediction models based on the SSA-LSSVM. The performance of the retrained model on the test set is shown in Table 10. For the prediction of AMT, the model trained with only these three features showed a significant reduction in performance in all aspects. However, for the prediction of the removal flow, the model with the downscaled data was the same as the model with the complete data, with no reduction in any indicators. Therefore, it can be stated that the feature ‘average milk yield’ contributes to the prediction of AMT but not to the prediction of the removal flow.

### 3.4. Potential Applications and Challenges

In this study, the machine learning prediction model was developed to predict the AMT and removal flow of individual cows, achieving satisfactory performance on the validation set. The method to implement the prediction of milking parameters for individual cows is different from some prediction studies on cow milking [31,40,41]. It is more dynamic, practical, and completely innovative, focusing on individual cows in real time (based only on the first 2 min of data during the milking).

The objective of this study was to improve milking efficiency by reducing milking time through precision milking parameter settings for individual cows. Its potential use is very promising, but some challenges exist in the application. The milking equipment currently used in dairy farms does not have the capability of adjusting milking parameters in real time. The integration of this research in milking systems could be a challenge to realizing the application of this research. In addition, since the milking is continuous, high demands are placed on the real-time nature of the prediction model. Relying on this study can promote more innovative and practical developments. For example, when the predicted results deviate significantly from the actual outcome, it might be possible to identify a cow with stress or health problems, and precise treatment of individual cows can be achieved, for example, by adjusting environmental parameters [42] and adjusting cow nutrition [43]. Therefore, its implementation could also serve as an early-warning technique for stress or illnesses.

## 4. Conclusions

This study collected a full set of data about milking operations on a dairy cattle farm located in Northern Italy. Somatic cell count, clinical mastitis, and teat-end score were also considered for evaluating the results on the udder health of cows. The experiment included a trial with the change in settings for the pulsation ratio and another trial for the change in settings of the detachment flow rate. With respect to the need of reducing the duration of milking operations, results are in line with literature findings, showing the benefits of a pulsation ratio set at 65:35 to reduce the milking time, although this involved higher hyperkeratosis. Concerning the increase in the detachment flow rate to 800 g/min, milk yield was reduced and SCC improved, although the teat scores worsened slightly.

However, to evaluate the possibility of automatically modifying the milking parameters during milking, based on cows’ specificities, the optimized SSA-LSSVM algorithm developed in this study achieved very interesting results in the prediction of the average milking time and of the removal flow rate of each cow. This model, if implemented on milking machines on farms, could permit automatically adjusting the parameters of the milking machine based on the first 2 min of milking of each cow. Therefore, it could represent a valuable improvement for milking operations in terms of duration, production, and health. In the future, the potentialities of technology, data science, and IoT will further increase and influence the sector in the continuous search for efficiency improvements. Moreover, the possibility of using algorithms that can be learned based on single cows’ characteristics will also improve the health and welfare needs of each farmed animal and will support the early warning of illnesses by monitoring the milk ejection.

## Figures and Tables

**Table 1:** Corrected least-square means (LSMeans), standard errors (S.E.s), and related statistical differences of Trial 1, where 2 pulsation ratios were tested. The control had the pulsation ratio set to 60:40, and the experiment had the pulsation ratio set to 65:35.

Parameter	Unit	Trial 1		S.E.	*p*	
		Puls60:40	Puls65:35			
AMT	min	8.42	7.97	0.09	0.0003	**
Yield	kg/session	18.8	19.2	0.30	0.2769	n.s.
Total Low Flow	min	1.10	1.40	0.07	0.0028	**
Peak Time	min	4.47	4.23	0.08	0.0409	*
Peak Flow	g/min	3684.4	3823.9	97.4	0.3145	n.s.
Removal Flow	g/min	860.6	880.5	14.8	0.3438	n.s.

Note: significance levels: n.s.: not significant; *: <0.05; **: <0.001.

**Table 2:** Corrected least-square means (LSMeans), standard errors (S.E.s), and related statistical differences of Trial 2, where 2 detachment flows were tested. The control had the detachment flow set to 600 g/min, and the experiment had the detachment flow set to 800 g/min.

Parameter	Unit	Trial 2		S.E.	*p*	
		Detach600	Detach800			
AMT	min	4.94	5.02	0.05	0.2793	n.s.
Yield	kg/session	18.5	17.8	0.24	0.0485	*
Total Low Flow	min	0.76	0.64	0.04	0.0387	*
Peak Time	min	2.27	2.28	0.04	0.8638	n.s.
Peak Flow	g/min	6432.2	6651.3	120.7	0.1973	n.s.
Removal Flow	g/min	1083.6	1213.9	24.9	0.0003	**

Note: significance levels: n.s.: not significant; *: <0.05; **: <0.001.

**Table 3:** Least-square means (LSM), standard error (S.E.), and statistical difference (*p*-value) of the somatic cell count in the 3 measurements (1–3) in chronological order for Trial 1.

Parameter	Trial 1		S.E.	*p*	
	Puls60:40	Puls65:35			
SCC_1	3.11	2.38	0.06	<0.0001	***
SCC_2	3.28	3.23	0.07	0.1946	n.s.
SCC_3	2.72	2.58	0.06	<0.0001	***

Note: significance levels: n.s.: not significant; ***: <0.0001.

**Table 4:** Least-square means (LSM), standard error (S.E.), and statistical difference (*p*-value) of the somatic cells count in the 3 measurements (1–3) in chronological order for Trial 2.

Parameter	Trial 2		S.E.	*p*	
	Detach600	Detach800			
SCC_1	2.66	2.73	0.03	0.0137	**
SCC_2	3.56	2.68	0.05	<0.0001	***
SCC_3	4.29	3.86	0.04	<0.0001	***

Note: significance levels: **: <0.001, ***: <0.0001.

**Table 5:** Teat scores of cows (TS-1 to TS-5) in chronological order, distinguished between front and back teats for Trial 1 and Trial 2. Cells background is colored with green for the low TS values and it becomes red when the TS values worsen (higher TS values).

Trial	Teats	Group	TS-1	TS-2	TS-3	TS-4	TS-5
Trial 1	Front	puls60:40	2.10	2.09	2.23	2.35	2.57
		puls65:35	2.23	2.29	2.33	2.43	2.65
	Back	puls60:40	2.03	2.11	2.11	2.27	2.39
		puls65:35	2.25	2.24	2.28	2.36	2.41
Trial 2	Front	detach600	2.03	1.98	2.04	2.07	1.97
		detach800	2.11	2.15	2.18	2.20	2.24
	Back	detach600	2.00	1.97	2.00	2.00	1.93
		detach800	2.08	2.11	2.09	2.14	2.13

**Table 6:** Least-square means (LSMs), standard error (S.E.), and statistical difference (*p*-value) of the teat-end scores (TS-1 to TS-5) in chronological order for Trial 1.

Parameter	Trial 1		S.E.	*p*	
	Puls60:40	Puls65:35			
TS_1	2.25	2.34	0.01	<0.0001	***
TS_2	2.33	2.46	0.02	<0.0001	***
TS_3	2.49	2.58	0.02	<0.0001	***
TS_4	2.44	2.57	0.02	<0.0001	***
TS_5	2.78	2.83	0.02	<0.0001	***

Note: significance levels: ***: <0.0001.

**Table 7:** Least-square means (LSM), standard error (S.E.), and statistical difference (*p*-value) of the teat-end scores (TS-1 to TS-5) in chronological order for Trial 2.

Parameter	Trial 2			*p*	
	Detach600	Detach800			
TS_1	2.09	2.10	0.01	0.0187	**
TS_2	1.99	2.11	0.01	<0.0001	***
TS_3	2.02	2.10	0.01	<0.0001	***
TS_4	2.10	2.17	0.01	<0.0001	***
TS_5	2.18	2.34	0.02	<0.0001	***

Note: significance levels: **: <0.001; ***: <0.0001.

**Table 8:** AMT prediction model performance.

Tested Models	Accuracy	Precision	Recall	F1 Score
SSA-LSSVM	0.92	0.97	0.94	0.95
LSSVM	0.90	0.96	0.93	0.94
Naive Bayes	0.82	0.83	0.96	0.89
Decision Tree	0.87	0.94	0.91	0.93
KNN	0.89	0.93	0.94	0.94
LDA	0.90	0.97	0.91	0.94

**Table 9:** Removal flow prediction model performance.

Tested Models	Accuracy	Precision	Recall	F1 Score
SSA-LSSVM	0.78	0.78	0.99	0.88
LSSVM	0.75	0.78	0.93	0.85
Naive Bayes	0.76	0.79	0.94	0.86
Decision Tree	0.64	0.79	0.73	0.76
KNN	0.67	0.79	0.80	0.79
LDA	0.58	0.80	0.61	0.69

**Table 10:** Performance of the SSA-LSSVM model based on reduced dimensional data.

		Accuracy	Precision	Recall	F1 Score
SSA-LSSVM	Trial 1	0.87	0.68	0.33	0.44
	Trial 2	0.78	0.79	0.99	0.88

## Data Availability

The data presented in this study are available on request from the corresponding author.
